# Transcriptome Profiling of Placenta through Pregnancy Reveals Dysregulation of Bile Acids Transport and Detoxification Function

**DOI:** 10.3390/ijms20174099

**Published:** 2019-08-22

**Authors:** Peng Wang, Yumo Song, Heju Zhong, Sen Lin, Xiaoling Zhang, Jian Li, Lianqiang Che, Bin Feng, Yan Lin, Shengyu Xu, Yong Zhuo, De Wu, Douglas G. Burrin, Zhengfeng Fang

**Affiliations:** 1 Key Laboratory for Animal Disease Resistance Nutrition of the Ministry of Education, Animal Nutrition Institute, Sichuan Agricultural University, Chengdu 611130, China; 2 USDA/ARS Children’s Nutrition Research Center, Section of Gastroenterology, Hepatology and Nutrition, Department of Pediatrics, Baylor College of Medicine, Houston, TX 77030, USA

**Keywords:** placenta, pregnancy, high-throughput RNA sequencing (RNA-Seq), bile acid transport, bile acid sulfation, long non-coding RNA (lncRNA)

## Abstract

Placenta performs the function of several adult organs for the fetus during intrauterine life. Because of the dramatic physiological and metabolic changes during pregnancy and the strong association between maternal metabolism and placental function, the possibility that variation in gene expression patterns during pregnancy might be linked to fetal health warrants investigation. Here, next-generation RNA sequencing was used to investigate the expression profile, including mRNAs and long non-coding RNAs (lncRNAs) of placentas on day 60 of gestation (G60), day 90 of gestation (G90), and on the farrowing day (L0) in pregnant swine. Bioinformatics analysis of differentially expressed mRNAs and lncRNAs consistently showed dysregulation of bile acids transport and detoxification as pregnancy progress. We found the differentially expressed mRNAs, particularly bile salt export pump (*ABCB11*), organic anion-transporting polypeptide 1A2 (*OATP1A2*), carbonic anhydrase II (*CA2*), Na^+^-HCO_3_^−^ cotransporter (*NBC1*), and hydroxysteroid sulfotransferases (*SULT2A1*) play an important role in bile acids transport and sulfation in placentas during pregnancy. We also found the potential regulation role of *ALDBSSCG0000000220* and *XLOC_1301271* on placental *SULT2A1*. These findings have uncovered a previously unclear function and its genetic basis for bile acids metabolism in developing placentas and have important implications for exploring the potential physiological and pathological pathway to improve fetal outcomes.

## 1. Introduction

Placenta is a temporary organ supporting the normal growth and development of the fetus during pregnancy. During this period, placenta is not only responsible for exchange of oxygen, iron, and substrate transfer such as sugar and amino acids, but also participates in proteins and peptide hormones synthesis and placental immunology [1,2]. Moreover, placenta also performs the function of several adult organs for the fetus during intrauterine life [3]. For example, toxic endogenous compounds including bile acids (BA) are transformed and eliminated by the hepatobiliary system with the collaboration of the kidney in adult [4]. However, the situation is different for the fetus during pregnancy. Owing to its immaturity, fetal liver can produce BA, but is not able to eliminate them into bile [5,6,7,8,9]. Placenta is the major organ responsible for fetal BA and biliary pigments transport. Then, the BA and biliary pigments of fetal origin are eliminated into feces and urine after biotransformation through maternal liver and, to a lesser extent, maternal kidney [9]. Noteworthy, several pregnancy-associated liver diseases are associated with impaired placental function, especially in intrahepatic cholestasis of pregnancy (ICP). Placental BA transport function is impaired in ICP patients and thus has adverse effect on fetal pregnancy outcomes through limiting fetal BA excretion [10,11,12]. Thus, maintaining proper function of placentas has great implication for fetal health [13].

Pregnancy is commonly associated with extensive physiological and metabolic changes to accommodate the growing needs of fetuses [14]. A typical example is the progressive increase of serum BA during pregnancy, especially in the second and the third trimester [15,16], and a subset of women with serum BA level above reference ranges (≥11 μmol/L) develop intrahepatic cholestasis of pregnancy (ICP) [17]. Studies in ICP patients and maternal cholestasis model during pregnancy have shown the impaired placental function, including the reduced fetal BA transport from fetuses to mother [18] and placental oxidative stress and apoptosis (19) [19]. Consistent with results in pregnant women [15], maternal BA metabolism in pregnant swine was dysregulated with the advance of pregnancy [8]. In addition, our recent study also indicated the impairment of placental BA transport function as pregnancy advanced, which further affected the BA homeostasis of fetuses [8]. However, the genetic and molecular basis for placenta to perform preprogrammed function, particularly BA metabolism, is largely undetermined as pregnancy advances. Next generation high-throughput RNA-Sequencing (RNA-Seq) has become a popular and comprehensively informative approach to study transcriptional changes of placentas [20,21,22]. Recent studies have revealed that lncRNAs participate in modulation of biological functions, through regulating gene expression during chromatin remodeling, transcription, and post-transcription, and thus could be a potential therapeutic target [23,24,25]. Therefore, our aim in this study was to identify the key mRNAs and lncRNAs, especially enriched in BA metabolism, of placentas with the advance of pregnancy in swine through RNA-Seq. Our data revealed the key genes responsible for dysregulation of BA transport and detoxification in placentas with the advance of pregnancy.

## 2. Results

### 2.1. RNA Sequencing and Identification of mRNAs in All Porcine Placentas

RNA-Seq methods were used to detect mRNA and lncRNA transcripts. RNA-Seq data showed an average of 109,932,040 raw reads and 106,071,702 clean reads per sample were obtained, and an average of 71.08% were mapped to a reference *sus scrofa* genome (Table S1). The percentage of reads mapped to the reference genome were similar between groups, suggesting that there were no sequencing biases in the data.

The expression levels were calculated and presented as a box plot of logarithmic transformed FPKM+1 values for each sample separately (Figure S1A), and the FPKM density distribution is shown in Figure S1B.

### 2.2. RNA Sequencing and Identification of Long Non-Coding RNAs (lncRNAs) in All Porcine Placentas

**Figure 1:**
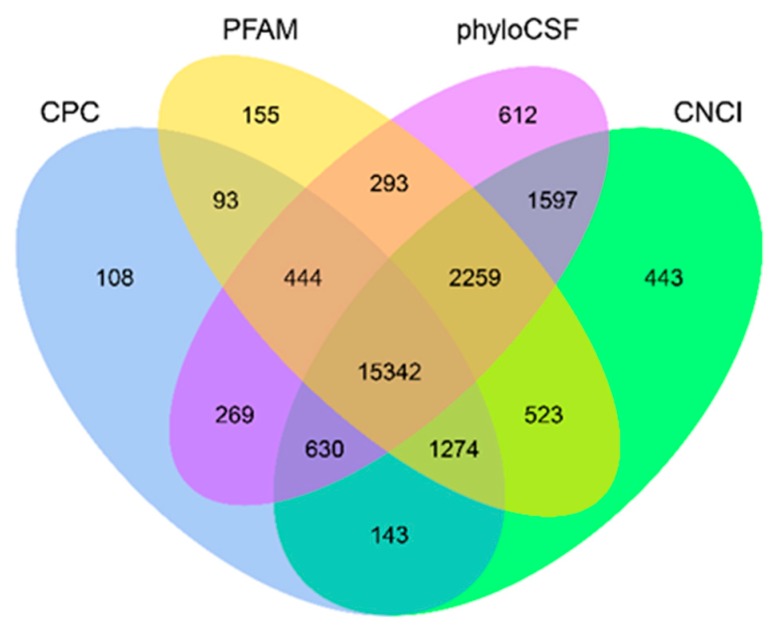
Screening of the candidate long non-coding RNAs (lncRNAs) in placenta transcriptome. Coding Potential Calculator (CPC), Coding-Non-Coding-Index (CNCI), Pfam-scan (PFAM) and phylogenetic codon substitution frequency (phyloCSF) were used to analyze the coding potential of lncRNAs. CPC, Coding Potential Calculator; PFAM, Pfamscan; PhyloCSF, phylogenetic codon substitution frequency; CNCI, Coding-Non-Coding-Index.

A total of 24,578 novel lncRNAs were assembled by Cufflinks and basic Scripture (Figure S2). As the identification of transcripts involved potential protein–coding fragments, Coding Potential Calculator (CPC), Pfam-scan (PFAM), phylogenetic codon substitution frequency (phyloCSF) and Coding-Non-Coding-Index (CNCI) were used to remove potential coding transcripts, and 15,342 putative lncRNAs were retained (Figure 1). The ALDB database was chosen as the lncRNA annotation reference, and 1220 lncRNAs were identified in the ALDB lncRNA annotation, indicating the presence of many novel lncRNAs (Figure S3).

### 2.3. Comparison of Features of mRNAs and Long Non-Coding RNAs (lncRNAs)

**Figure 2:**
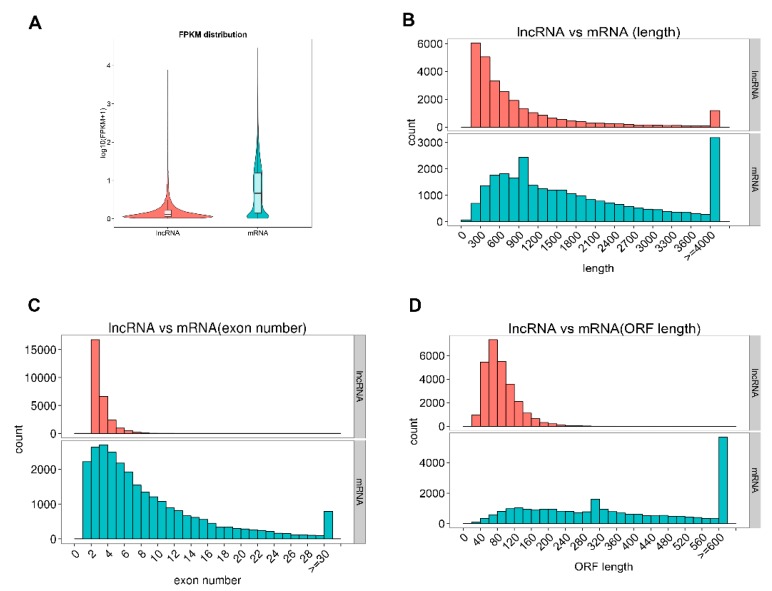
Comparison of expression levels and genomic feature of predicted long non-coding RNAs (lncRNAs) and mRNAs in pregnant swine placentas. (**A**) The expression levels (log_10_ (FPKM+1)) of mRNAs and lncRNAs; The (**B**) length, (**C**) exon number, (**D**) Open reading frame (ORF) length of mRNAs and lncRNAs, respectively.

In this study, we characterized the basic feature of the lncRNAs and compared them with mRNAs. Our results showed that average lncRNAs expression level was lower than mRNAs and the identified lncRNAs were shorter in length than mRNAs, and they tended to have fewer exons (Figure 2A–C). The average length and exon of lncRNAs were 1045.84 bp long and 2.64 exons, respectively, while the mRNAs were 1982.69 bp long and 8.71 exons, respectively. Furthermore, the identified lncRNAs tended to be shorter in ORF length than mRNAs and most lncRNAs were less conserved than mRNAs (Figure 2D, Figure S4).

### 2.4. Placentas at Different Time of Pregnancy Have Distinct Gene Expression Profiles and Cluster Separately

**Figure 3:**
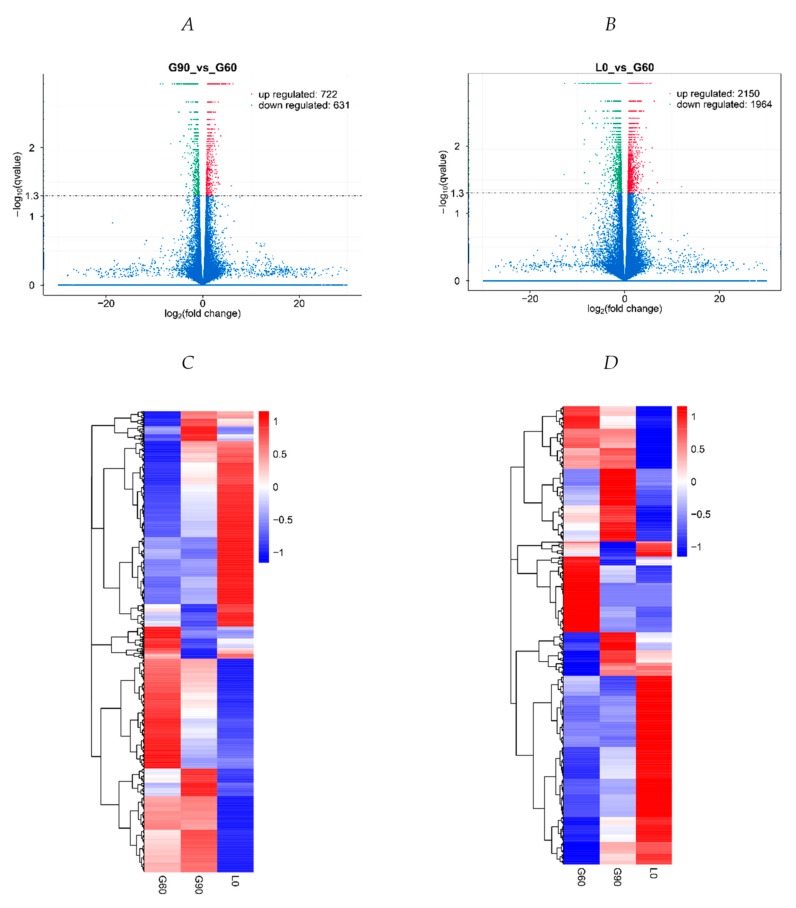
Transcriptome profile of RNA-seq data at G60, G90, and L0. (**A**) A volcano plot of differentially expressed transcripts, including mRNAs and long non-coding RNAs (lncRNAs), between G60 and G90, and (**B**) between G60 and L0. (**C**) Unsupervised hierarchical clustering of the expression profile of significant genes of mRNAs, and (**D**) lncRNAs among G60, G90, and L0.

Compared with G60 placentas, 1079 mRNA transcripts (552 up-regulated and 527 down-regulated) and 274 lncRNA transcripts (170 up-regulated and 104 down-regulated) were differentially expressed in G90 placentas (*p* < 0.01, corrected *p* < 0.05) (Figure 3A, Table S2), whereas 3291 mRNA transcripts (1602 up-regulated and 1689 down-regulated) and 823 lncRNA transcripts (548 up-regulated and 275 down-regulated) were differentially expressed in L0 gilts (*p* < 0.005, corrected *p* < 0.05) (Figure 3B, Table S3). In unsupervised hierarchical clustering analysis, heat maps were generated using the differentially expressed lncRNAs and mRNAs, respectively, and they clearly self-segregated into G60, G90, and L0 (Figure 3C,D). These results reflect distinct lncRNAs and mRNAs expression profiles between different stages of pregnancy. Six mRNAs and three novel lncRNAs were chosen for quantitative RT-PCR to confirm the reliability of RNA-Seq (Figure S5). The relative expression of mRNAs and lncRNAs determined by qRT-PCR showed consistent changes with RNA-seq results.

### 2.5. Dysfunction of Bile Acid Transport and Detoxification Are the Central Characteristics with the Advance of Pregnancy

**Figure 4:**
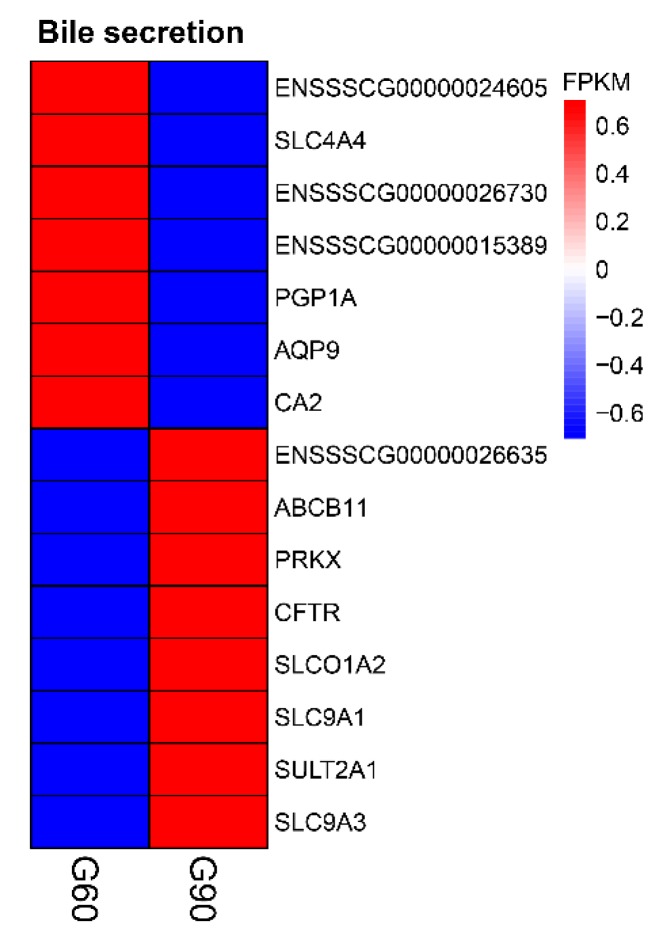
Unsupervised hierarchical clustering of the expression profile of significant genes enriched in bile secretion between G60 and G90 placentas.

To ascertain the functions of the differentially expressed mRNAs and connections among them, we performed GO term and KEGG pathway enrichment analysis between groups. In GO analysis of the differentially expressed mRNAs between G60 and G90, the significantly over represented terms are shown in Table S4, including apical plasma membrane, apical part of cell, cell surface, and extracellular region in cellular component; transporter activity and hydrolase activity in molecular function; biological process, metabolic process, transport, single-organism transport, transmembrane transport, bicarbonate transport, regulation of transport, and cellular process in biological process. KEGG pathway analysis on G90 vs. G60 showed that bile secretion and complement and coagulation cascades were the most enriched terms (*p* < 0.01, corrected *p* < 0.05) (Figure 4, Figure S6).

**Figure 5:**
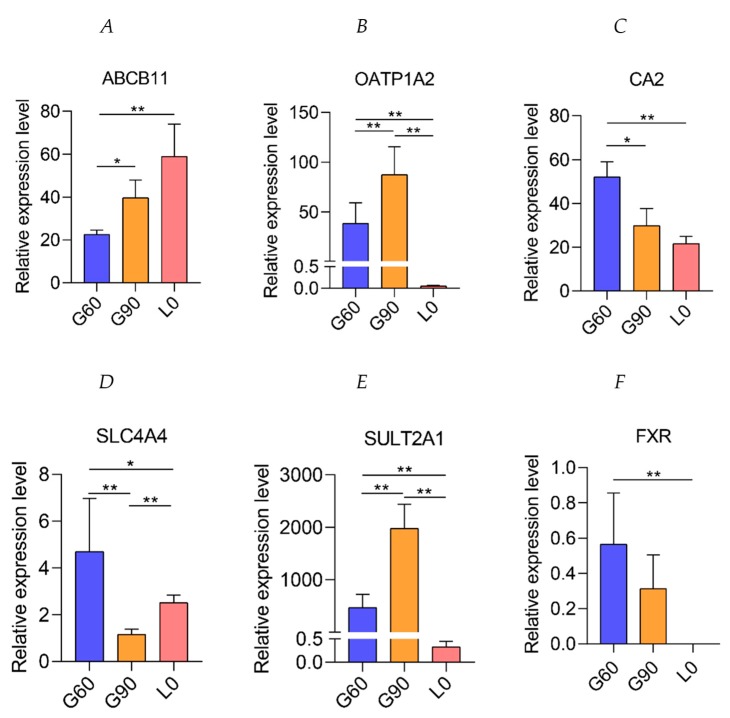
The differentially expressed genes involved in bile secretion at G60, G90, and L0 placentas. (**A**) The relative expression levels of *ABCB11*, (**B**) organic anion-transporting polypeptide 1A2 (*OATP1A2)*, (**C**) carbonic anhydrase II (*CA2)*, (**D**) *SLC4A4*, (**E**) hydroxysteroid sulfotransferases (*SULT2A1)*, and (**F**) *FXR* determined by RNA-Seq at G60, G90, and L0. Data are shown as means ± SE, * *p* < 0.05, ** *p* < 0.01.

In the enriched bile secretion pathway, a total of 15 genes were significantly (*p* < 0.01, corrected *p* < 0.05) altered in G90 placentas compared with G60 placentas (Figure 4). The expression of bile salt export pump (*ABCB11*, also known as *BSEP*), located in the apical membrane of human and rat hepatocytes and reported to export BA in ATP-dependent manner [26,27], were higher at G90 and L0 than at G60 (Figure 5A). The expression of organic anion-transporting polypeptide 1A2 (*SLCO1A2*, also known as *OATP-A*), mediating hepatocellular uptake of organic anions, including conjugated BA and estrone-3-sulfate, accompanied by bicarbonate efflux [28,29,30], was higher at G90 than at G60, while lower at L0 than at G60 (Figure 5B). It was noteworthy that carbonic anhydrase II (*CA2*), catalyzing the conversion of CO_2_ to the bicarbonate ion and protons [31], and Na^+^-HCO_3_^−^ cotransporter (*NBC1*, also known as *SLC4A4*) (Figure 5C,D), which participates in bicarbonate production and uptake [32,33], were significantly down-regulated at G90 and L0 compared with that at G60, suggesting impairment of BA exchange between fetal and maternal units. These observations suggested the availability of bicarbonate might be limited, which may lead to the metabolic derangement in patients with cholestatic liver diseases [34] and thus may impair the ability of placentas to transport fetal BA into maternal circulation.

**Figure 6:**
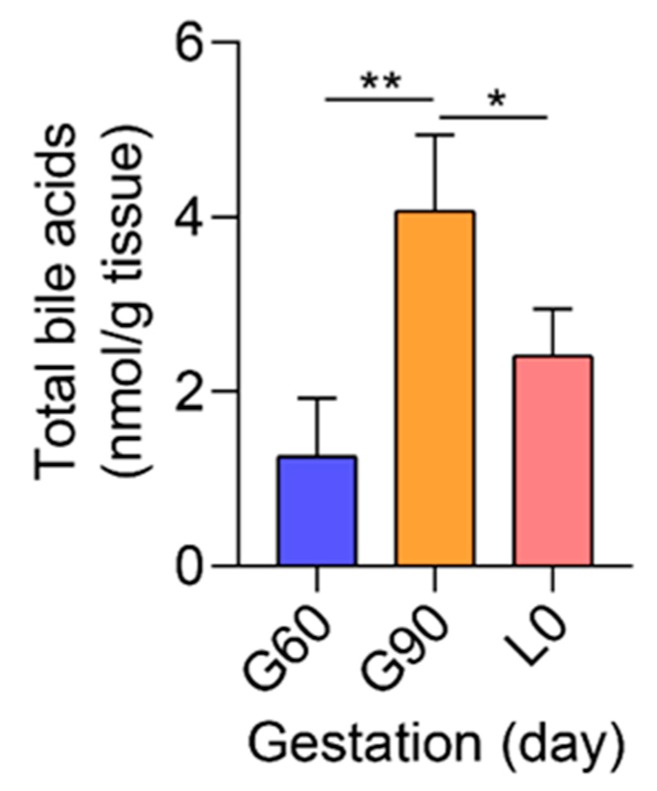
The total bile acids (TBA) of placentas used for RNA-Seq at G60, G90, and L0. *n* = 3–4/group. Data are shown as means ± SE, * *p* < 0.05, ** *p* < 0.01.

Sulfation is an important pathway for the detoxification and elimination of BA during cholestatic liver diseases [35,36,37]. As a member of cytosolic sulfotransferases enzymes (SULTs), hydroxysteroid sulfotransferases (*SULT2A1*) mediates sulfo-conjugation of BA [38], leading to increased water solubility and thus facilitating BA elimination [35]. Placental expression of *SULT2A1* was higher at G90 than at G60, indicating the improved detoxification function of placentas, but was significantly decreased at L0 compared with that at G60 or G90 (Figure 5E). *FXR*, also known as *NR1H4*, can be activated by BA [39], especially CDCA, DCA, and LCA, and plays an important role in *SULT2A1* expression in liver [40] and a protective role in maternal cholestasis induced damage and oxidative stress [41]. Unexpectedly, the expression of *FXR* was not significantly different between G60 and G90 placentas and reached undetectable level in L0 placentas (Figure 5F). Consistent with KEGG pathway analysis, the TBA concentrations of placentas used for RNA-Seq were significantly increased from G60 to G90, and not significantly different between G60 and L0 (Figure 6). Further investigation showed that maternal serum TBA had a trend pattern similar with that in placentas (Figure S7).

**Figure 7:**
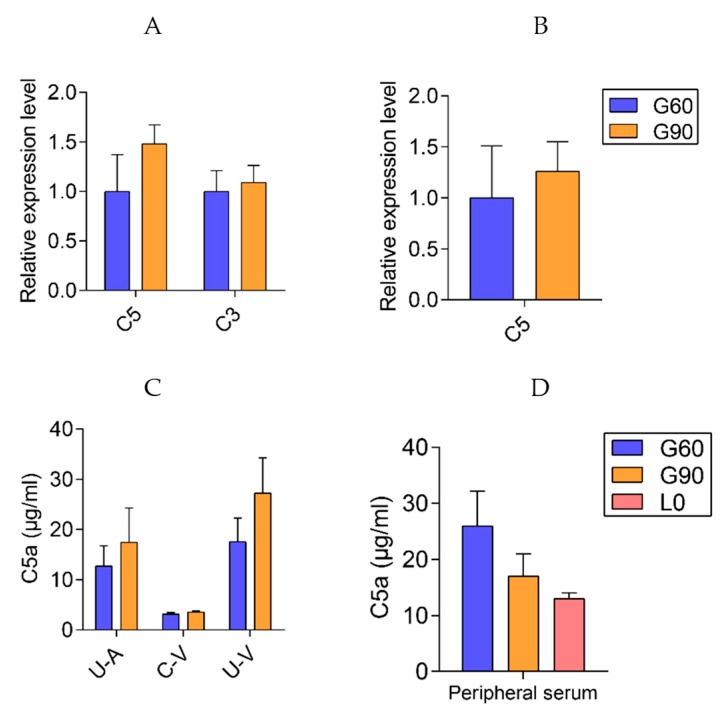
The complement system in livers and placentas. The expression of hepatic complement *C3* and *C5* (**A**) and placental complement *C5* (**B**) between G60 and G90 determined by qRT-PCR, *n* = 3–6/group. (**C**) The concentrations of complement C5a in U-A, U-V, C-V serum between G60 and G90, *n* = 5–6/group. (**D**)The concentrations of complement C5a in maternal peripheral serum, *n* = 10/group. U-A, uterine artery, C-V, umbilical cord vein, U-V, uterine vein. Data are shown as means ± SE.

In the enriched complement and coagulation cascades pathway, a total of 16 genes were differentially expressed (*p* < 0.01, corrected *p* < 0.05) in G90 placentas compared with that in G60 placentas (Figure 4). Remarkably, placental *C5aR1* (*C5R1*, *CD88*) expression at G90 was twice that at G60, and the higher *C5aR1* expression was maintained till L0 when maternal TBA levels restored to the levels at G60 (Figure 4, Figure S5). As the main source of C5a, the ligand of C5aR1, *C5* expression did not differ between G90 and G60 placentas (Figure 7B). Placental *C3* is an upstream of C5 in complement system, and, interestingly, the expression of *ENSSSCG00000030385*, complement C3 precursor showed a lower expression at G90 than at G60 (Figure 4). The upregulation of complement factor H (*CFH*), which promotes dissociation of the C3 convertase enzyme [42] and protects against complement attack [43], might account for the decreased expression of *C3* in G90 placentas. Expression of high levels of complement regulatory proteins including membrane cofactor decay accelerating factor (*DAF*, also known as *CD55*) was reported to be critical for the trophoblast to prevent excessive complement activation in the placentas [44]. It would appear that the higher placental expression of *CD55* at G90 than at G60 might be an adaptive response to activated complement system. In support of this notion, the gene *F2*, encoding for thrombin protein which is capable of generating the complement activation product C5a in the absence of C3 [45], showed a lower expression at G90 than at G60 placentas. Collectively, placental mRNA levels of genes on which we focused seemed not applicable to explain the up-regulated *C5aR1*. Given that 80–90% of the complement proteins are produced by the liver [46], we further tested the *C3* and *C5* expression in liver tissues and C5a concentrations in maternal peripheral serum and U-A, C-V, and U-C serum. No differences were observed in hepatic *C3* and *C5* expression and in serum C5a concentrations with the advance of gestation stage (Figure 7A,C,D).

Analyzing the differentially expressed mRNAs between L0 and G60, we found no significantly enriched terms in KEGG pathway (Figure S8A), although there existed plenty of GO enrichment terms, including regulation of transport, ATP synthesis coupled proton transport, transport, regulation of intracellular transport, sulfur compound metabolic process in biological process, and sulfur compound binding and catalytic activity in molecular function (Table S5).

### 2.6. Prediction of Long Non-Coded RNAs (LncRNAs) Function

**Figure 8:**
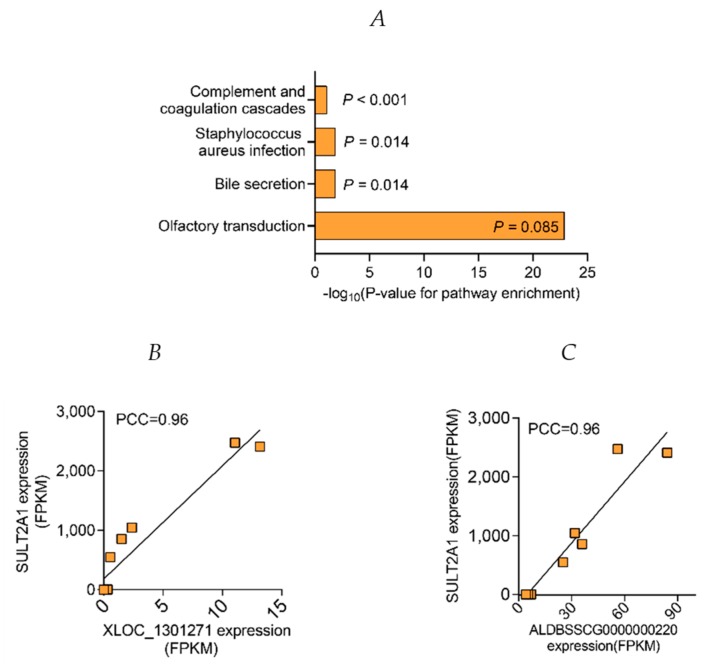
The significantly enriched Kyoto Encyclopendia of Genes and Genomes (KEGG) pathway of predicted trans-regulated targeted protein-coding genes of differentially expressed lncRNAs between G60 and G90. (**A**) The significantly enriched KEGG pathway of significantly differentially expressed mRNAs between G60 and G90. (**B**) A scatterplot of *XLOC_1301271* and *SULT2A1* expression levels in individual placental samples determined by RNA-Seq. (**C**) A scatterplot of *ALDBSSCG0000000220* and *SULT2A1* expression levels in individual placental samples determined by RNA-Seq. PCC, Pearson Correlation Coefficient; *n* = 3–4/group.

Recent studies have demonstrated that lncRNAs can regulate the expression of nearby and remote protein-coding genes in cis and trans, respectively [47,48]. The genomic feature of lncRNAs are shown in Figure S2. We performed the GO enrichment and KEGG pathway analysis of cis-mRNAs to predict the function of dysregulated lncRNAs between G90 and G60, no significant enrichment (corrected *p* > 0.05) was found since the number of cis-mRNAs was relatively small. There was also no significant enrichment (corrected *p* > 0.05) in GO analysis between L0 and G60, except “membrane-bound organelle”, which belongs to cellular component terms. We performed GO analysis on their trans-regulated target lncRNAs in two comparison groups. In GO terms, the significantly over-represented terms are shown in Tables S6 and S7 (*p* < 0.001, corrected *p* < 0.05). Although the existence of many common GO terms, no significantly enriched KEGG pathway was found between L0 and G60 (Figure S8B). We performed KEGG pathway analysis on their trans-regulated target lncRNAs between G90 and G60 (Figure 8). The significantly over-represented terms (*p* < 0.01, corrected *p* < 0.05) between G90 and G60 were olfactory transduction, bile secretion, staphylococcus aureus infection, and complement and coagulation cascades (corrected *p* = 0.08). The predicted function “bile secretion” and “complement and coagulation cascades” were same with mRNA, indicating the potential strong connection and interaction between lncRNAs and mRNAs. Remarkably, the up-regulated *XLOC_1301271* and *ALDBSSCG0000000220* were matched with *SULT2A1* (Figure 7B,C). The increased expression of *XLOC_1301271* and *ALDBSSCG0000000220* might affect expression of *SULT2A1* and therefore be involved in the BA metabolism in placentas. Moreover, there were some significantly enriched KEGG pathways different from mRNA results, including olfactory transduction and staphylococcus aureus infection (corrected *p* < 0.05). A recent study indicates that BA are natural ligands of olfactory systems [49]. However, the detailed relationship between these remained to be elucidated yet.

## 3. Discussion

Raised maternal serum BA is one of the most common phenomena among humans, rodents, and pigs during pregnancy [8,50,51,52]. High levels of maternal serum BA have been implicated in high levels of fetal BA through impairing transplacental export of BA from fetal into maternal system, especially when the fetal liver was able to synthesis BA, but not able to eliminate it, thus resulting in high risk of fetal death [10,53,54]. Previous reviews have summarized the hepatobiliary-like excretory role of placenta during pregnancy [55], however, the detailed placental function and its genetic basis during pregnancy are poorly understood. We here show novel findings in gilts demonstrating the dysregulated BA transport and metabolism and its genetic basis during pregnancy through mRNAs and lncRNAs analysis of placental function in developing placentas.

Consistent with impaired BA transport system of placentas in ICP patients and complete obstructive cholestasis in rats [12,18], our data showed that BA transport function of placentas was also impaired with the advance of pregnancy. Transporters, export pumps, and nuclear receptors involved in the liver excretory function may play a similar role in the placentas [56]. Studies in isolated membrane vesicles of human and rat placentas [57,58] have revealed the ATP-dependent transport system for BA across the TPMa plays an important role in the control of the overall fetal-maternal BA traffic. The multidrug resistance protein 2 (*MRP2*, also known as *ABCC2*), transports BA [59] and bilirubin glucuronides in ATP-dependent manner [60], and multidrug resistance protein 3 (*MRP3*, also known as *ABCC3*), also transports BA in ATP-dependent manner [61]. Consistent with the existence of *ABCC2* and *ABCC3* in rat placenta [55], our data showed the expression of *ABCC2* and *ABCC3* from G60 to L0, but their expression were not significantly different among G60, G90 and L0 (Tables S2 and S3). On the contrary, the expression of *ABCB11* was continuously increased from G60 to L0, and might be the main BA transport carrier located in the TPMa during pregnancy [55]. Moreover, in response to raised maternal BA at G90 compared with that at G60, the equivalent maternal BA input into placenta and output from placenta to mother [8] further verified the BA transport role of *ABCB11* in TPMa.

Studies in isolated membrane vesicles of human and rat placentas [62,63] have revealed that bicarbonate plays a critical role for BA exchange in the TPMb. In this study, *SLC01A2*, mediating hepatocellular uptake of organic anions including conjugated BA, accompanied by bicarbonate efflux [28,29,30], may be the main gene located in the TPMb and responsible for fetal BA excretion during pregnancy [9,55]. We further revealed the expression of *SLC01A2*, an orthologue of human *OATP1A2* [64], was highly correlated with maternal serum and placental TBA, as indicated by the expression of *SLC01A2* which was up-regulated from G60 to G90 when the maternal and placental TBA were increased, whereas was down-regulated from G90 to L0 when the maternal and placental TBA were decreased. These observations were consistent with the report that *OATP* was up-regulated by BA [28]. Interestingly, the expression of *SLC01A2* was not regulated by fetal serum BA, as fetal serum BA was continuously increased from G60 to L0. This observation may be explained by our previous results that placental TBA was of maternal origin, but not of fetal origin [8]. Unexpectedly, placental bicarbonate concentration was likely to decrease with the advance of pregnancy, which could be explained by two aspects. Firstly, placental expression of *CA2* and *SLC4A4*, responsible for bicarbonate production and uptake, respectively, were continuously decreased from G60 to L0. Secondly, carbonic anhydrase (CA), including bovine CA and human CA1 and CA2, were potently inhibited by bile salts [34] and showed BA species dependent effect [65,66,67]. In consideration of that CO_2_ diffuses across placenta predominantly in the molecular form rather than as bicarbonate ion [33] and the bicarbonate in mother was higher than in fetal unit in normal pregnancy [68], the decreased bicarbonate in placenta may limit BA exchange between fetus and mother in the TPMb.

In addition, our data also revealed the regulated BA metabolism in developing placentas. In humans, the hydrosteroid SULT (SULT2) family is comprised of two subfamilies, SULT2A1 and SULT2B1. SULT2A1 mediates sulfo-conjugation of endogenous molecules, including androgens, estrogens, and glucocorticoids, as well as BA, and thus facilitating BA detoxification and elimination [35], whereas SULT2B1 does not work in this way [38]. In this study, the expression of *SULT2A1* was significantly increased from G60 to G90, whereas it decreased from G90 to L0. Maternal serum sulfated BA being higher at G90 than at G60, and not significantly different between G90 and L0, further verified the BA sulfation function of placental *SULT2A1*, especially when the expression of *SULT2A1* in liver was not significantly different [8]. Furthermore, recent studies have revealed that lncRNAs, such as *lnc-HC* and *MEG3*, participate in BA metabolism, through regulating cholesterol 7α-hydroxylase (*CYP7A1*) and small heterodimer partner (*SHP*) [69,70]. In this study, the high consistency between function of dysregulated mRNAs and predicted functions of dysregulated lncRNAs suggests that lncRNAs play a critical role in regulating the expression of mRNAs, especially the potential regulation role of *ALDBSSCG0000000220* and *XLOC_1301271* on placental *SULT2A1*.

Bile acids have also been reported to induce placental inflammation by activating G protein-coupled bile acid receptor 1(*Gpbar1*)/ nuclear factor kappa B (*NF-κB*) [71], and placental damage, oxidative stress and apoptosis through tumor necrosis factor (*TNF*, also known as *TNFα*) and *FXR* [41,72]. The expression of these genes was not significantly different among G60, G90, and L0 (Tables S2 and S3), implying that the gestation stage-dependent increase of placental bile acids may be not sufficient to evoke placental inflammation and oxidative stress.

In summary, genes encoding proteins responsible for placental BA transport and detoxification were dysregulated as pregnancy advanced. The high consistency between mRNAs and predicted functions of dysregulated lncRNAs implies that lncRNAs may play an important role in regulating the expression of mRNAs during pregnancy. These findings have uncovered a previously unclear function and its genetic basis in developing placentas and have important implications for exploring the potential physiological and pathological pathway to improve fetal outcomes.

## 4. Materials and Methods

### 4.1. Experimental Design and Sampling

A total of 10 placentas were sampled as described [73] from 10 LY (Landrace × Yorkshire) gilts on day 60 of gestation (G60), day 90 of gestation (G90), and the farrowing day (L0), respectively (*n* = 3, 3, and 4, respectively). Briefly, the placentas were collected 5 cm away from the umbilical cord and selected from fetuses with average body weight. The number of fetuses at G60, G90, and L0 is shown in Table S8. In consideration of that, serum BA levels were greatly affected by the postprandial time, which reached the peak levels at around 2 h after a meal and returned to base-line levels within 4 h in human [74,75], whereas it reached the peak levels at around 2 h after a meal and returned to base-line levels within 8 h in sows in our unpublished data, placentas, maternal livers, blood of uterine artery (U-A), uterine vein (U-V), and umbilical cord vein (C-V) were sampled after 8-h fasting when sows were laparotomized and under deep isoflurane-induced anesthesia at G60 and G90, respectively. Placental samples and peripheral blood samples were also collected at L0 during delivery. The peripheral blood samples of mothers were collected via catheters implanted at cephalic vein as described [76] after 12-h fasting or via ear vein when catheters did not work. All samples were immediately stored at −80 °C until analysis. The nutrient requirement of sows in every stage were determined according to the National Swine Nutrition Guide (NSNG, 2010, Ames, IA, USA) and the diet composition and feeding regimen were the same as we previously described [8]. The protocol of this study was approved by the Animal Care and Use Committee of Animal Nutrition Institute, Sichuan Agricultural University, and was carried out in accordance with the National Research Council’s Guide for the Care and Use of Laboratory Animals, Chinese Order No.676 of the State Council, date (1 March 2017).

### 4.2. Total Bile Acids

The total BA (TBA) were measured by the enzymatic cycling method assay kits in 7020 automatic biochemical analyzer (Hitachi, Tokyo, Japan) as described.

### 4.3. RNA Sequencing and Data Analysis of the Porcine Placentas

Total RNAs were isolated and quality controlled. Ribosomal RNA (rRNA) and rRNA free residue were removed by Epicentre Ribo-zero™ rRNA Removal Kit (Epicentre, Madison, WI, USA) and ethanol precipitation, respectively. RNA-Seq was performed on an Illumina Hiseq 2000 platform and 150 bp paired-end reads were generated according to Illumina’s protocol. Sequencing libraries were generated using the rRNA-depleted RNA by NEBNext^®^ Ultra™ Directional RNA Library Prep Kit for Illumina^®^ (NEB, Ipswich, MA, USA) following manufacturer’s recommendations. Products were purified by AMPure XP system (Beckman Coulter, Orange County, CA, USA) and library quality was assessed on the Agilent Bioanalyzer 2100 system (Agilent, Santa Clara, CA, USA). The data were analyzed and annotated by a pipeline developed in-house. Briefly, high quality clean reads were aligned to the reference genome using TopHat v2.0.9 (Washington, DC, USA). The mapped read were assembled by both Scripture (beta2, Cambridge, MA, USA) [77] and Cufflinks (v2.1.1, College Park, MD, USA) [78]. The candidate set of lncRNAs were filtered by CNCI (Coding-Non-Coding-Index) (v2, Beijing, China), CPC (Coding Potential Calculator), (0.9-r2, Beijing, China), Pfam Scan (v1.3, Hinxton, UK) and PhyloCSF (phylogenetic codon substitution frequency) (v20121028, Cambridge, MA, USA) tools. The differential expression analysis of mRNAs and lncRNAs was performed by Cufdiff based on the negative binomial distribution [78], with an *p*-adjust < 0.05 were assigned as differentially expressed. GOseq R package (Release 2.12, Melbourne, Australia) and KOBAS software (v2.0, Beijing, China) were used to perform the Gene Ontology (GO) and KEGG enrichment analysis of differential expression genes or lncRNAs target gene, respectively.

### 4.4. Real-Time RT-PCR

Total RNA was extracted from the frozen samples using the RNAiso Plus reagent (Takara Bio, Ltd., Dalian, China) according to the manufacturer’s specifications. The complementary DNA (cDNA) was synthesized using a reverse transcription kit (Takara Bio, Ltd., Dalian, China) and stored at 4 °C prior to real-time quantitative PCR (q PCR). Real-time PCR was performed on a QuanStudio^TM^ 6 Flex Real-Time PCR system (Applied Biosystems, Foster City, CA, USA) to quantify mRNAs or lncRNA expression with a commercial SYBR Green kit (Takara Bio, Inc., Dalian, China). Specific primers were designed using the software of primer BLAST from NCBI (Table S9). Amplification efficiency was controlled by the use of an internal control (β-actin). Relative quantification of target mRNAs or lncRNAs were calculated and normalized to β-actin expression. The gene expression levels of different samples was compared by the 2^−ΔΔ*C*T^ method [79,80].

### 4.5. Measurement of C5a

The C5a level of uterine artery, umbilical cord vein, uterine vein, and maternal vein serum were measured using the Elisa assay kits (Nanjing JianCheng Bioengineering Institute, Nanjing, China) following the manufacturer’s instruction.

### 4.6. Data Analysis

All data were analyzed by using the SAS statistical package (V9.4, SAS Institute Inc., Cary, NC, USA), except for the RNA-seq result. All values were expressed as means ± SE. Significant differences were analyzed by PROC GLM and least significant difference test or using the NPAR1WAY procedure of SAS 9.4 test for samples that were not normally distributed or homogeneous variances. *p* < 0.05 was considered statistically significant.

## Supplementary Materials

Supplementary materials can be found at https://www.mdpi.com/1422-0067/20/17/4099/s1.

## Abbreviations

ICP	Intrahepatic cholestasis of pregnancy
RNA-Seq	RNA sequencing
BA	Bile acid
LncRNA	Long non-coding RNA
ABCB11/BSEP	Bile salt export pump
OATP1A2/SLCO1A2	Organic anion-transporting polypeptide 1A2
TPMa	Apical plasma membrane of the trophoblast
TPMb	Basal plasma membrane of the trophoblast
NBC1/SLC4A4	Na^+^-HCO_3_^−^ cotransporter
SULT2A1	Hydroxysteroid sulfotransferases
CA2	Carbonate anhydrase 2
FXR/NR1H4	Farnesoid X receptor
