# Supplementary material for: Transcriptome Profiling of Placenta through Pregnancy Reveals Dysregulation of Bile Acids Transport and Detoxification Function
Source: Int J Mol Sci. 2019 Aug 22;20(17):4099. doi: 10.3390/ijms20174099 (PMC6747679; doi:10.3390/ijms20174099)
Supplement: Supplementary file 1 [file ijms-20-04099-s001.zip › ijms-535692-for final sup/Supplementary Figure revised .pdf]

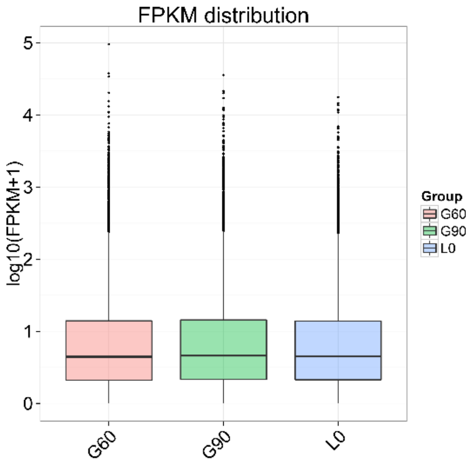

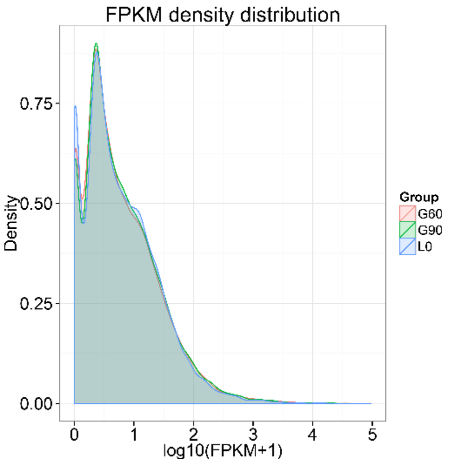

(A) (B)

Figure S1 Transcripts expression level distribution of each placenta sample. (A) A box plot of FPKM distribution with different gestation stage on the horizontal axis and Log_{10} (FPKM+1) on the vertical axis. (B) Density plot of expression distribution with Log_{10} (FPKM + 1) on the horizontal axis and density on the vertical axis.

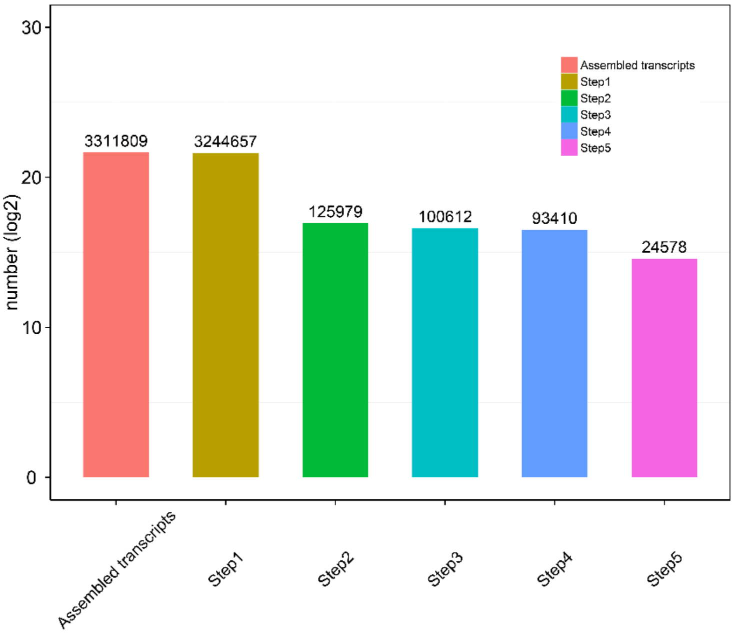

Figure S2 24,578 non-coding lncRNAs were selected by using Cufflinks and Scripture.

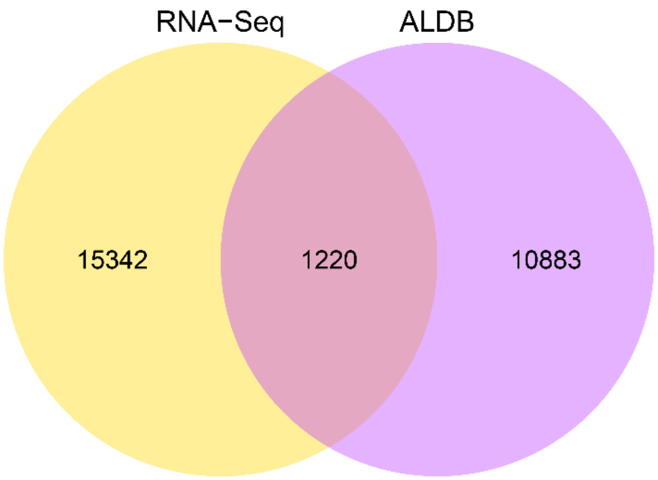

Figure S3 Screening of the known and novel lncRNAs in placenta transcriptome. Identification known and novel lncRNAs from ALDB.

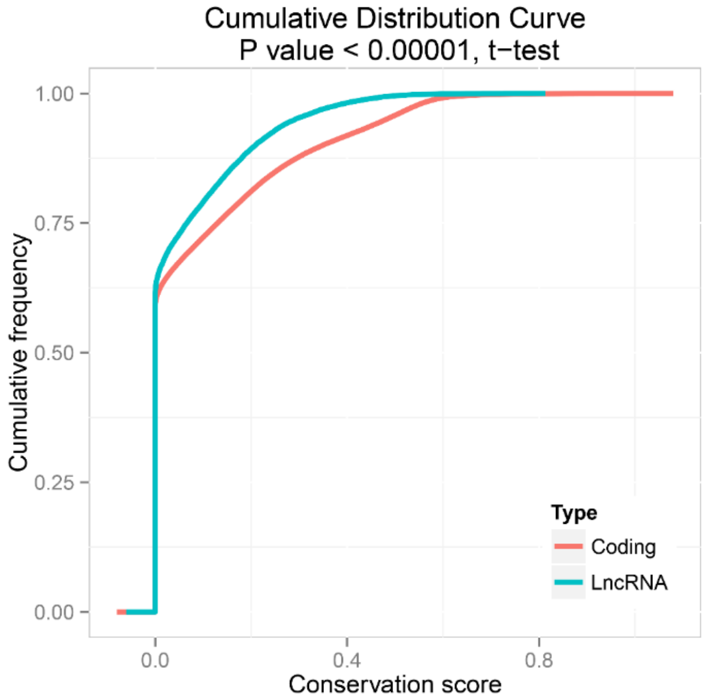

Figure S4 The conservation of the sequence in mRNAs and LncRNAs were evaluated using phyloP (http://compgen.bscb.cornell.edu/phast/).

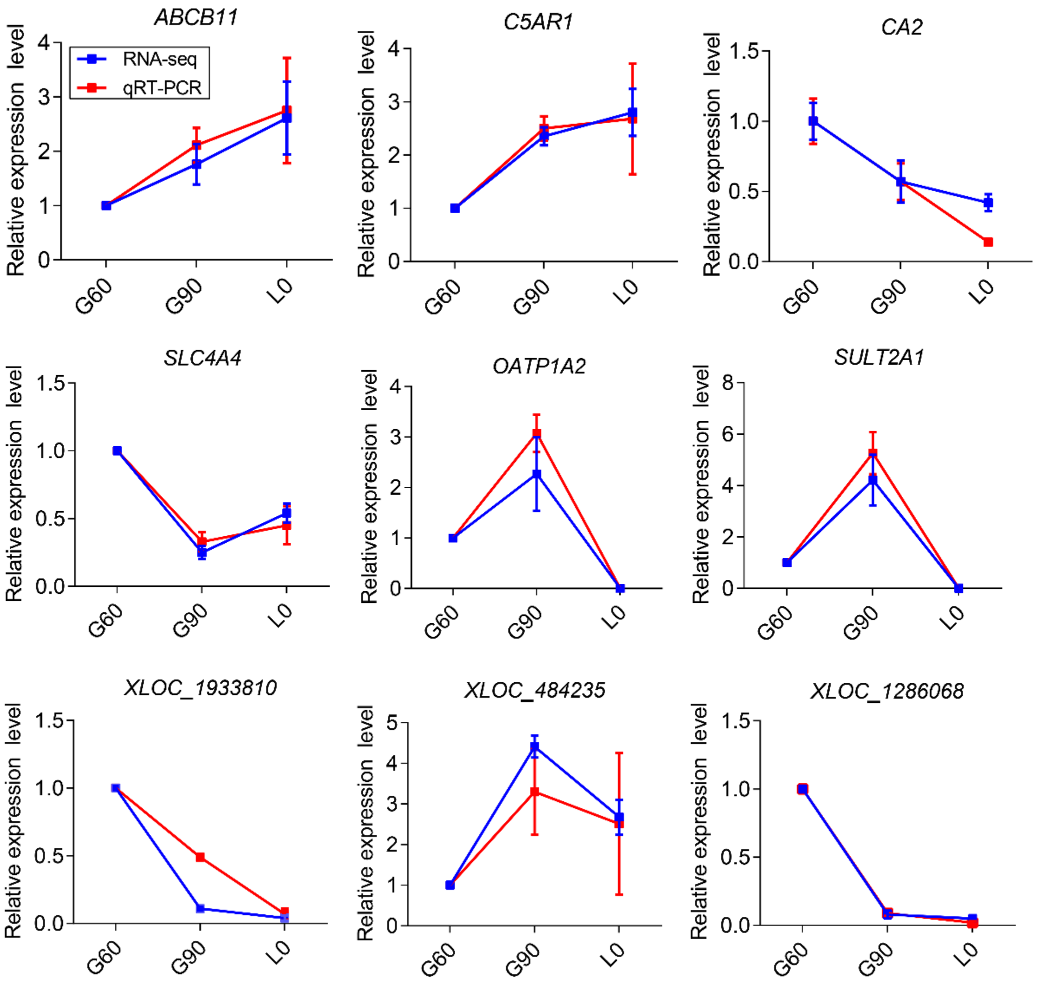

Figure S5 Confirmation of the expression patterns of mRNAs and lncRNAs using qRT-PCR. Six mRNA and three novel lncRNA were chosen for quantitative RT-PCR to confirm the reliability of RNA-Seq. Data are shown as means ± SE.

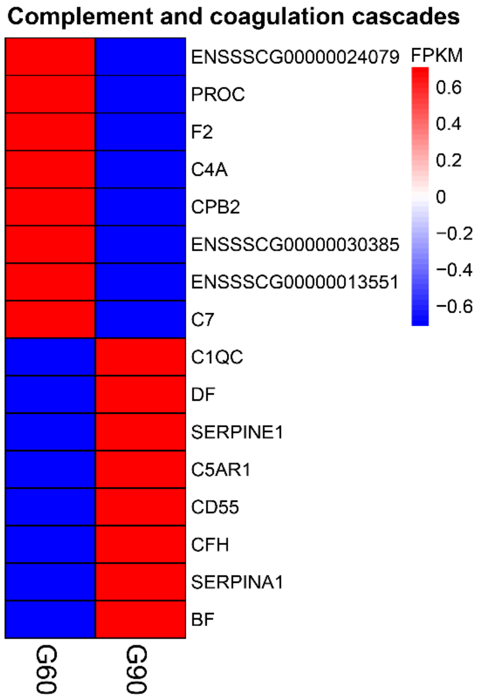

Figure S6 Unsupervised hierarchical clustering of the expression profile of significant genes enriched in bile secretion between G60 and G90.

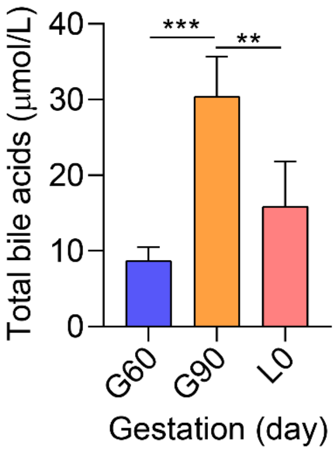

Figure S7 The dynamic change of TBA in maternal serum at G60, G90 and L0, respectively (n=10/group). Data are shown as means ± SE, $^{*}P$ <0.05, $^{**}P$ <0.01, $^{***}P$<0.001.

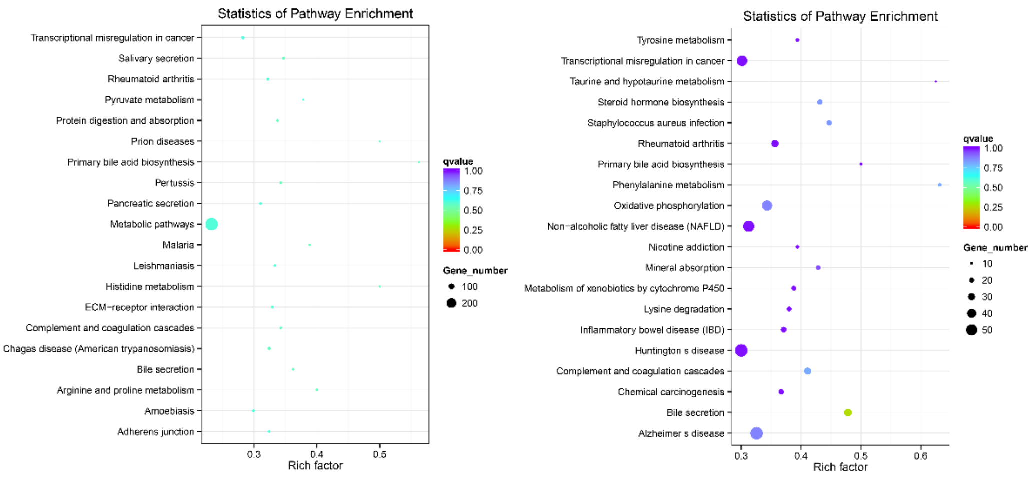

(A) (B)

Figure S8 The KEGG pathway analysis of dysregulated mRNAs and predicted trans-regulated targeted mRNAs of differentially expressed lncRNAs. (A) The top 20 over-represented KEGG pathways of dysregulated mRNAs, and (B) predicted trans-regulated targeted mRNAs of differentially expressed lncRNAs between G60 and L0.
