# Supplementary material for: Transcriptome Profiling of Placenta through Pregnancy Reveals Dysregulation of Bile Acids Transport and Detoxification Function
Source: Int J Mol Sci. 2019 Aug 22;20(17):4099. doi: 10.3390/ijms20174099 (PMC6747679; doi:10.3390/ijms20174099)
Supplement: Supplementary file 1 [file ijms-20-04099-s001.zip › ijms-535692-for final sup/Supplementary Table S1 .pdf]

Table S1. Quality control and alignment statistics of RNA-seq reads to sus scrofa genome

Sample ID	G60_1	G60_2	G60_3	G90_1	G90_2	G90_3	L0_1	L0_2	L0_3	L0_4
Raw reads	91320404	1.09E+08	1.1E+08	1.25E+08	1E+08	1.1E+08	1.09E+08	1.19E+08	1.07E+08	1.19E+08
Clean reads	87508166	1.05E+08	1.05E+08	1.2E+08	96208856	1.05E+08	1.06E+08	1.16E+08	1.05E+08	1.15E+08
Total mapped	69003108 (78.85%)	83894900 (79.78%)	83109017 (79%)	97025366 (80.78%)	76484130 (79.5%)	84176986 (80.22%)	83243390 (78.34%)	90397034 (77.74%)	82724715 (79.09%)	88074917 (76.48%)
Multiple mapped	11076425 (12.66%)	13452462 (12.79%)	14799743 (14.07%)	13623914 (11.34%)	18444907 (19.17%)	12458069 (11.87%)	10671131 (10.04%)	9019371 (7.76%)	8779608 (8.39%)	13860220 (12.03%)
Uniquely mapped	57926683 (66.2%)	70442438 (66.99%)	68309274 (64.93%)	83401452 (69.44%)	58039223 (60.33%)	71718917 (68.35%)	72572259 (68.3%)	81377663 (69.99%)	73945107 (70.7%)	74214697 (64.44%)
Reads map to '+'	28778127 (32.89%)	35007414 (33.29%)	33931403 (32.25%)	41523765 (34.57%)	28821620 (29.96%)	35632932 (33.96%)	35862630 (33.75%)	40053938 (34.45%)	36797288 (35.18%)	36478109 (31.67%)
Reads map to '-'	29148556 (33.31%)	35435024 (33.7%)	34377871 (32.68%)	41877687 (34.87%)	29217603 (30.37%)	36085985 (34.39%)	36709629 (34.55%)	41323725 (35.54%)	37147819 (35.52%)	37736588 (32.77%)
Non-splice reads	39765473 (45.44%)	47794218 (45.45%)	44987126 (42.76%)	59176485 (49.27%)	43825497 (45.55%)	51121867 (48.72%)	56268743 (52.95%)	64024867 (55.06%)	55965146 (53.51%)	55289997 (48.01%)
Splice reads	18161210 (20.75%)	22648220 (21.54%)	23322148 (22.17%)	24224967 (20.17%)	14213726 (14.77%)	20597050 (19.63%)	16303516 (15.34%)	17352796 (14.92%)	17979961 (17.19%)	18924700 (16.43%)
