# Supplementary material for: Transcriptome Profiling of Placenta through Pregnancy Reveals Dysregulation of Bile Acids Transport and Detoxification Function
Source: Int J Mol Sci. 2019 Aug 22;20(17):4099. doi: 10.3390/ijms20174099 (PMC6747679; doi:10.3390/ijms20174099)
Supplement: Supplementary file 1 [file ijms-20-04099-s001.zip › ijms-535692-for final sup/Supplementary Table S8,9.pdf]

Table S8. The number of fetuses at G60, G90 and L0

Item	G60	G90	L0
Litter size	13.67	12.67	11.25
Live litter size	13.67	12.67	9.75

Table S9. Primer sets for real-time RT-PCR analysis

Gene	Forward（5'-3'）	Reverse（5'-3'）	Accession number
β-actin	AGAGCAAGAGAGGCATCCTG	CACGCAGCTCGTTGTAGAAG	XM_003124280.5
BSEP/ABCB11	GCCTGACCACGAGCATCT	AGGTCAGTTTCCAACCCTGAT	XM_003133457.5
SULT2A1	CCATGCGAGACAAGGAGAAC	CATGACCTGGAAGGAGCTGT	NM_001037150.1
C5aR1	TGGACTATGGTCGAGACGGT	ACAGATGGAGAGGGTGACCA	NM_001244215.1
C3	AGGCATCGTATCCTCTGGGA	ACATGGTCACCACCGACAAG	NM_214009.1
C5	ACTTGGTGACCTTCGACGTG	ACCCCTTGGGTCCAGAGTAA	NM_001001646.1
CA2	CTTTGACCCTCGTGACCTCC	CCATCGGGTGTTCAGGTTCA	XM_001927805.2
OATP1A2/SLC01A2	TGACACTGGATCGGTGAACA	AGGAATGGCAGTGAGCACAT	NM_001256595.1
XLOC_1286068	AGCATCTGACTGAAGGACCAG	AGGGTCAGACTCAACATTTCCA	
XLOC_484235	CAGCCACCATCCACACTGAC	GCCAGGCAAGGACTTGTACG	
XLOC_1933810	TAGCCCATCTGCTACCCAGC	AGCATGGCCAACAGTAACCAA	
